# Long-Lasting Effects of GSPE on Ileal *GLP-1R* Gene Expression Are Associated with a Hypomethylation of the *GLP-1R* Promoter in Female Wistar Rats

**DOI:** 10.3390/biom9120865

**Published:** 2019-12-12

**Authors:** Iris Ginés, Katherine Gil-Cardoso, Claudio D’Addario, Anastasia Falconi, Fabio Bellia, M Teresa Blay, Ximena Terra, Anna Ardévol, Montserrat Pinent, Raúl Beltrán-Debón

**Affiliations:** 1 MoBioFood Research Group, Universitat Rovira i Virgili, Departament de Bioquímica i Biotecnologia, c/Marcel·lí Domingo 1, 43007 Tarragona, Spain; iris.gines@urv.cat (I.G.); katherine.gil@urv.cat (K.G.-C.); mteresa.blay@urv.cat (M.T.B.); ximena.terra@urv.cat (X.T.); anna.ardevol@urv.cat (A.A.); raul.beltran@urv.cat (R.B.-D.); 2 Faculty of Bioscience and Technology for Food, Agriculture and Environment, University of Teramo, Via Renato Balzarini 1, 64100 Teramo, Italy; cdaddario@unite.it (C.D.); anastasiafalconi@live.it (A.F.); fabio.bellia91@gmail.com (F.B.)

**Keywords:** flavanols, proanthocyanidins, epigenetics, methylation, intestine, *GLP-1R*, SP1

## Abstract

Flavonoids have been shown to modulate *GLP-1* in obesity. *GLP-1* induces some of its effects through the intestinal *GLP-1* receptor (*GLP-1R*), though no data exist on how flavonoids affect this receptor. Here, we examine how a dose of grape seed proanthocyanidin extract (GSPE) with anti-obesity activity affects intestinal *GLP-1R* and analyze whether epigenetics play a role in the long-lasting effects of GSPE. We found that 10-day GSPE administration prior to the cafeteria diet upregulated *GLP-1R* mRNA in the ileum 17 weeks after the GSPE treatment. This was associated with a hypomethylation of the *GLP-1R* promoter near the region where the SP1 transcription factor binds. In the colon, the cafeteria diet upregulated *GLP-1R* without showing any GSPE effect. In conclusion, we have identified long-lasting GSPE effects on *GLP-1R* gene expression in the ileum that are partly mediated by hypomethylation at the gene promoter and may affect the SP1 binding factor.

## 1. Introduction

Obesity is one of the most prevalent diseases worldwide. Its development is influenced by numerous factors, including energy balance alterations, genetic predisposition, gut microbiota disorders, imbalance between oxidative stress and antioxidant defense, environmental factors, endocrine imbalance, etc., all of which can lead to metabolic and epigenetic alterations [1]. One widely studied approach for treating metabolic disorders caused by obesity is the use of natural bioactive compounds. Flavonoids in particular are reported to act against obesity by modulating numerous metabolic pathways such as the lipid and glucose management in peripheral tissues [2,3]. For example, they have been shown to act as lipolytic agents inhibiting the lipase activity [4], to limit the formation of white adipose [5,6], to activate energy-consuming pathways [7,8], and to act in the gastrointestinal (GI) tract [9,10,11].

Recent studies by our research group have shown that a dose of 500 mg/kg BW of a grape seed extract rich in proanthocyanidins (GSPE) has long-lasting effects on reducing body weight, adiposity and modulating the respiratory quotient (RQ) in Wistar female rats that have been subjected to 17 weeks of cafeteria diet [12]. These effects suggest that epigenetic mechanisms may be involved, though this has not been confirmed. Boqué et al. showed that an apple polyphenol extract can induce epigenetic changes in adipose tissue, which may explain some of the anti-obesogenic effects observed [13]. Flavonoids modulate epigenetic mechanisms [13,14]. DNA methylation, the most widely studied epigenetic mechanism, occurs when a methyl group is added to the C5 position of cytosine (5mC), predominantly at CpG sites, via the DNA methyltransferases (DNMTs) [15]. It has been suggested that some flavanols, such as catechin, epicatechin, and epigallocatechin gallate, reverse DNA hypermethylation and that this reversal is mediated by the inhibition of DNMT1 [16]. As some flavonoids are also known to inhibit histone acetyltransferase and histone deacetylase, they may also interfere in histone remodeling [14,17].

The targets of flavonoids when they exert their anti-obesogenic effects are diverse. One of the first targets of flavonoids after ingestion is the GI wall, thus having plenty of opportunities to exert their effects by acting along the GI tract. In particular, flavanols have been shown to limit energy absorption by influencing the intestinal processes involved in the digestion and absorption of energy compounds [4] and to modulate inflammation and barrier properties [18]. They can also alter bacterial populations in the gut [19] as well as modulate gastrointestinal (GI) motility [20] and gastric emptying [21]. Serrano et al. showed that a dose of 500 mg/kg bw of a grape-seed proanthocyanidin extract (GSPE) was able to limit food intake [22], modify enteroendocrine hormone secretions [23], and decrease gastric emptying in female rats, thus inducing a satiating effect [24]. Moreover, the authors suggested that GSPE acts on food intake and body weight through vagal *GLP-1* receptor (*GLP-1R*) activation on the hypothalamic centre of food intake control and *GLP-1* production in the intestine [25]. Similarly, puerarin, a dietary isoflavone, improves glucose homeostasis in obese diabetic mice and protects pancreatic β-cell survival by mechanisms that involve the activation of *GLP-1R* signaling and downstream targets [26].

*GLP-1R* is expressed in the small intestine and colon, specifically in the myenteric neural cells [27] and smooth muscle cells [28]. Some effects of *GLP-1*, such as the modulation of gastric emptying and gastrointestinal motility, are thought to be mediated through interaction with intestinal *GLP-1R* [27,28,29]. However, no data are available regarding the effect of flavonoids on intestinal *GLP-1R*. In this paper, we therefore study the effects of GSPE on intestinal *GLP-1R* at a dose previously shown to have long-lasting anti-obesity activity and analyze whether GSPE exerts an epigenetic modulation.

## 2. Materials and Methods

### 2.1. Proanthocyanidin Extract

The grape seed proanthocyanidin extract (GSPE) was provided by Les Dérivés Résiniques et Terpéniques (Dax, France). According to the manufacturer, the GSPE composition of the extract used in this study (Batch number 124029) contained: monomers of flavan-3-ols (21.3%), dimers (17.4%), trimers (16.3%), tetramers (13.3%), and oligomers (5–13 units; 31.7%) of proanthocyanidins. A detailed analysis of the monomeric to trimeric structures can be found in the study by Margalef and col [30].

### 2.2. Animal Experiments

Female rats weighing 240–270 g were purchased from Charles River Laboratories (Barcelona, Spain). After one week of adaptation, they were individually caged in animal quarters at 22 °C with a 12-h light/12-h dark cycle and fed ad libitum with a standard chow diet (Panlab 04, Barcelona, Spain) and tap water. As previously described [12], the rats were randomly distributed into experimental groups (*n* = 7–10/group) and fed a standard chow diet ad libitum until the end of the experiment. The control group (STD) received only the standard chow diet. The other groups, in addition to this diet, received a cafeteria diet as the model for a high fat/high sucrose diet and/or a GSPE supplement at different moments along the experiment. The STD group and the cafeteria group (CAF) received an oral gavage of tap water as a vehicle together with the chow diet and cafeteria diet respectively. The preventive treatment group (PRE) received an oral dose of 500 mg GSPE/Kg for 10 days before starting the cafeteria diet. The simultaneous intermittent treatment-CAF (SIT) group received an five-days oral dose of 500 mg GSPE/Kg together with the cafeteria diet every other week, and the corrective treatment (CORR) group received an oral dose of 500 mg GSPE/Kg daily during the final two weeks of the long-term cafeteria intervention (Figure S1).

The cafeteria diet consisted of bacon, sausages, biscuits with paté, carrots, muffins, and sugared milk, which induced voluntary hyperphagia [12]. This diet was offered ad libitum every day to the animals for 17 weeks. GSPE was dissolved in water and force-fed orally to the animals at 6 pm for each treatment at a volume of 500 µL one hour after all the available food had been removed. Animals that were not fed GSPE received water as a vehicle.

At the end of the study, the animals fasted for 1–4 h, were anaesthetized with sodic pentobarbital (70 mg/kg body weight) provided by Fagron Iberica (Barcelona, Spain), and exsanguinated from the abdominal aorta. Intestinal segments from the duodenum, jejunum, ileum, and proximal colon were immediately frozen in liquid nitrogen and stored at –80 °C for further analysis.

All procedures were approved by the Experimental Animal Ethics Committee of the Universitat Rovira i Virgili. (Code: 0152S/4655/2015)

### 2.3. Quantitative Real-Time RT-PCR Analysis

Total RNA was extracted using Trizol (Ambion, USA) and trichloromethane-ethanol (Panreac, Barcelona, Spain), and purified using a Qiagen RNAeasy kit (Qiagen, Hilden, Germany). The cDNA was generated using the High Capacity cDNA Reverse Transcription Kit (Applied Biosystems, Waltham, USA). Quantitative PCR amplification was performed using a specific TaqMan probe (Applied Biosystems, Waltham, USA): Rn00562406_m1 for *GLP-1* receptor and Rn00562293_m1 for proglucagon (*Gcg*), the gene encoding for *GLP-1*. The relative expression of each gene was compared with the control group using the 2^-ΔΔCt^ method, with *PPIA* (Rn00690933_m1), as reference.

### 2.4. Analysis of DNA Methylation

Genomic DNA was extracted from the ileum using the TRIzol Reagent (Life Technologies, Ambion, Austin, TX, USA) and from the colon using a DNeasy Blood and Tissue Kit (Qiagen, Hilden, Germany). The DNA underwent bisulfite modifications using a commercially available modification kit (Zymo Research, Irvine, CA, USA).

DNA methylation was assessed by pyrosequencing. Bisulfite-treated DNA was amplified using a PyroMark PCR Kit (Qiagen, Hilden, Germany) in accordance with the manufacturer’s protocol. The polymerase chain reaction conditions were as follows: 95 °C for 15 min, followed by 45 cycles of 94 °C for 30 s, 56 °C for 30 s, 72 °C for 30 s, and 72 °C for 10 min. Polymerase chain reaction products were verified by agarose electrophoresis. Pyrosequencing methylation analysis was conducted using the PyroMark Q24 (Qiagen, Hilden, Germany). The level of methylation was analyzed using PyroMark Q24 ID version 1.0.9 software (Qiagen), which calculates the methylation percentage mC/(mC + C) (where mC is methylated cytosine and C is unmethylated cytosine) for each CpG site and allows quantitative comparisons. The primer set sequences used for pyrosequencing were those that presented most CpG islands in the maximum number of pair-bases permitted by the PyroMark Q24 machine (Table 1).

Nucleotide sequences of the *GLP-1R* gene upstream from its transcription start site (TSS) were obtained from the EMBL-EBI (http://www.ebi.ac.uk/) and NCBI databases (https://www.ncbi.nlm.nih.gov/). For the *GLP-1R* gene, the promoter region studied corresponds to an intragenic region in humans and mice according to EMBL-EBI and NCBI databases. Predicted transcription factor binding sites within the studied DNA regions were obtained from the ALGGEN PROMO (http://alggen.lsi.upc.es/cgi-bin/promo_v3/promo/promoinit.cgi?dirDB=TF_8.3) website.

### 2.5. Statistical Analysis

Our results are expressed as mean ± standard error of the mean (SEM). One-way ANOVA was used to compare the treatments. *p*-values < 0.05 were considered to be statistically significant. These calculations were performed using XL-Stat 2017.01 (Addinsoft, Barcelona, Spain) software.

Spearman’s correlation coefficient was used to test for correlations between the methylation of the *GLP-1* promoter and the cecal SCFA of the animals. *p*-values < 0.05 were considered statistically significant. These calculations were performed using XL-Stat 2017 software.

## 3. Results

### 3.1. GSPE Has Long-Lasting Effects on GLP-1R Gene Expression in the Ileum in Rats under a Cafeteria Diet

**Figure 1:**
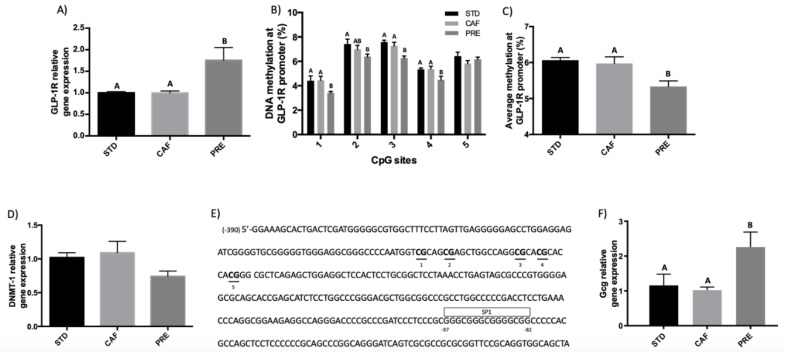
Effects of a 10-days preventive treatment of grape seed proanthocyanidin extract (GSPE) on (**A**) *GLP-1* receptor gene expression, (**B**) DNA methylation on five CpG sites of a region of the *GLP-1R* promoter, (**C**) average DNA methylation from the CpG sites of a region of *GLP-1R* promoter, (**D**) *DNMT-1* relative gene expression, (**E**) localization of the CpG sites (numbered 1–5) and of the putative binding site for the indicated transcription factor (framing) in the region flanking the rat *GLP-1R* gene transcription start site (+1) studied, and (**F**) *GgG* relative gene expression in the ileum. The data are the mean ± standard error of the mean (S.E.M.; *n* = 7–10) One-way ANOVA *p* < 0.05 was used to compare differences between the groups, obtained by a DMS posthoc test and defined by different letters.

First, we checked whether a 10-day pre-treatment of 500 mg/bw GSPE followed by a 17-week cafeteria diet (PRE) was able to induce changes in *GLP-1R* gene expression. Figure 1A shows that in the ileum *GLP-1R* gene expression was up-regulated 17 weeks after the final dose of GSPE. To check whether this long-lasting effect of GSPE was related to epigenetic mechanisms, we used pyrosequencing analyses to evaluate the methylation of the CpG islands present in a region of *GLP-1R* promoter. In agreement with the higher *GLP-1R* gene expression, Figure 1B,C show that, 17 weeks after the final GSPE dose, the methylation of the promoter in positions 1, 3, 4, and the average methylation of the CpG sites decreased. Indeed, a Spearman’s correlation test between the results of *GLP-1R* gene expression and the methylations of its promoter revealed a negative correlation for all groups between the gene expression and position 4 of the CpG islands (*p* = 0.063), position that presented a decreased methylation in the preventive treatment. We then checked the expression of *DNMT-1*, which is the methyltransferase responsible for maintaining the DNA methylation. Our results showed that, in comparison with the CAF group, there was a tendency for the mRNA levels of *DNMT-1* to decrease in the group that received the preventive treatment (*p* = 0.096; Figure 1D).

Moreover, in silico analysis with a web-based tool (ALGGEN PROMO) helped to predict the putative sites for the binding of transcription factors around positions –390 and +94 of the *GLP-1R* promoter. As we can see in Figure 1E, the study showed that in the region between positions –97 and –82, located near the promoter region of the methylation study, there was a putative SP1 transcription binding site.

Finally, to determine whether the long-lasting effects of GSPE extended not only to *GLP-1R* but also to its ligand hormone, we analyzed the effect of the pre-treatment with GSPE on *GLP-1* gene expression. Figure 1F shows that 17 weeks after the final dose of GSPE, *Gcg* gene expression, which corresponds to the gene that encodes for *GLP-1*, was also up-regulated compared to the CAF group. We aimed to study the DNA methylation pattern of *GLP-1* but observed that the *GLP-1* promoter region was devoid of CpG (based on a query of up to 5 kb and including exon 1 and intron 1). Moreover, as the effect of CpG DNA methylation on CpG-poor promoters is not well characterized, we did not study DNA methylation at the *GLP-1* promoter region.

### 3.2. Hypomethylation of GLP-1R Promoter Participates in the Regulation of GLP-1R Gene Expression after Short-Term Corrective GSPE Treatment in the Ileum

**Figure 2:**
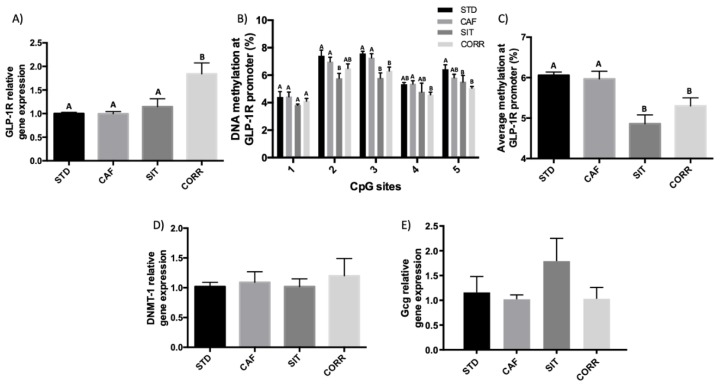
Effects of a 17 weeks simultaneous intermittent treatment (SIT) and a 15-days corrective treatment (CORR) of GSPE on (**A**) the *GLP-1* receptor, (**B**) DNA methylation on five CpG sites of a region of *GLP-1R* promoter, (**C**) average DNA methylation from the CpG sites of a region of *GLP-1R* promoter, (**D**) *DNMT-1* relative gene expression, and (**E**) *GLP-1* relative gene expression in the ileum. The data are the mean ± standard error of the mean (S.E.M.; *n* = 7–10) One-way ANOVA *p* < 0.05 was used to compare differences between the groups, obtained by a DMS posthoc test and defined by different letters.

We analyzed the effects of the same dose of GSPE administered both as a short-term corrective treatment at the end of the cafeteria diet (CORR) and for a long period throughout the cafeteria diet (SIT). GSPE also upregulated the *GLP-1R* gene expression (Figure 2A) in CORR, but not in SIT. As with the PRE group, to determine whether epigenetics were involved in this effect, we checked the methylation pattern for the same region of the *GLP-1R* promoter. Figure 2B,C show that the GSPE up-regulation in the CORR group was accompanied by a decrease in methylation in positions 3, 4, 5, and the average of methylations in the region of the promoter studied. Moreover, although the SIT treatment did not affect the *GLP-1R* gene expression, the SIT animals also presented a hypomethylation of the *GLP-1R* promoter in positions 2, 3, 5, and the average of methylation in the region of the promoter studied. With these treatments, GSPE led to no differences in *DNMT-1* mRNA (Figure 2D) and *GLP-1* relative gene expression (Figure 2E).

### 3.3. GSPE Effects in the Ileum Do not Extend to the Colon

**Figure 3:**
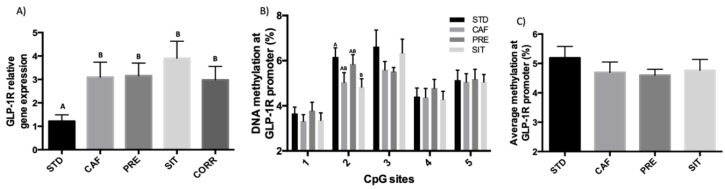
Effects of different GSPE treatments on the (**A**) *GLP-1* receptor. (**B**) DNA methylation on five CpG sites of a region of the *GLP-1R* promoter. (**C**) Average DNA methylation from the CpG sites of a region of the *GLP-1R* promoter. The data are the mean ± standard error of the mean (S.E.M.; *n* = 7–10) One-way ANOVA *p* < 0.05 was used to compare differences between the groups, obtained by a DMS posthoc test and defined by different letters.

In the colon, the long-term CAF diet significantly increased the expression of *GLP-1R* in comparison with the STD diet. However, in this case the GSPE treatments mentioned earlier did not modify this expression (Figure 3A). Similarly, GSPE led to no methylation changes in the *GLP1-R* promoter compared to the CAF group (Figure 3C). GSPE pre-treatment also led to no changes in *GLP-1* gene expression in the colon (CAF: 4.7 ± 0.4; PRE: 4.6 ± 0.2), though it did increase mRNA expression in SIT and CORR [31].

One of the clear differences between the ileum and the colon is the quality and quantity of colonic microbiota. Earlier we showed that the CAF group was able to significantly decrease butyric acid content and tended to increase propionic and isobutyric acid content. Moreover, the CORR treatment significantly reduced butyric acid content in comparison with CAF, while the SIT treatment did not lead to any significative changes [31]. Here we observed that the PRE group also showed no significative differences compared to the CAF group. However, to evaluate the possible effect of these changes on the methylation of the *GLP-1R* promotor and microbiota, we used Spearman’s correlation with the data from all the treatments tested to check whether any associations were found between the various CpG sites analyzed in the ileum (Table 2) and colon (Table 3) and the cecal short chain fatty acids measured at the end of the experiment (Supplementary Materials Table S1). In the ileum, we observed that some short chain fatty acids presented positive correlations with several positions of the CpG sites studied: butyric acid correlated positively with positions 4 and 5, valeric acid correlated positively with position 4, and succinic acid correlated positively with positions 2, 4, 5, and the average methylated CpG sites found in the *GLP-1R* promoter.

In the colon (Table 3), on the other hand, correlation with butyric acid was almost lost, while the correlation with succinic acid remained. Significant correlations also appeared between positions 4, 5, and average methylated CpG sites and acetic acid.

## 4. Discussion

In previous studies, we found that some GSPE anti-obesogenic effects were maintained when GSPE was administered preventively for only 10 days and before the administration of the cafeteria diet [12]. *GLP-1* acts not only as an enteroendocrine hormone but also on the gastrointestinal tract, where it modulates gastric emptying and gut motility [29,32], thus also possibly contributing to weight loss [33]. These effects have also been attributed to *GLP-1R* expressed in the small intestine and colon in the myenteric neural cells [27] and smooth muscle [28]. We therefore decided to check whether a 10-day preventive treatment with GSPE was able to modulate *GLP-1R* in both the ileum and the colon and whether these long-lasting effects were partly modulated by epigenetics. We also compared this preventive treatment with another preventive treatment administered intermittently during the cafeteria diet (SIT) and with a corrective treatment (CORR) administered at the end of the cafeteria diet.

Our results show that GSPE has long-lasting effects on ileal *GLP-1R* gene expression. Previous studies suggested epigenetics as a target for grape seed proanthocyanidins, thus altering the expression of various genes [34,35]. In our case, epigenetics seems to be a possible explanation for these long-term GSPE effects since we found that 10-day pre-treatment with GSPE induced hypomethylation of a region of the *GLP-1R* promoter that persisted for several weeks after the GSPE treatment and was consistent with the increased gene expression observed in this group. In addition, the hypomethylation of the *GLP-1R* promoter was also observed right after 15 days of GSPE treatment at the end of the cafeteria diet (CORR) and when the treatment was extended in simultaneous fashion throughout cafeteria feeding (SIT). In this case, the CORR treatment correlates with the gene expression of the gen that encodes for *GLP-1*, while the SIT treatment does not. Although we do not observe changes on the methylation pattern of the region of the promoter that we study, we do not discard other epigenetic mechanisms involved with the effects of GSPE on the SIT group. Moreover, there are plenty of biochemical mechanisms by which GSPE can act, and depending on the moment of its administration, different mechanisms might be activated. For example, GSPE has been demonstrated to modulate different kind of miRNAs [36,37], as well as histone deacetylases [35,38]. Apart from the epigenetic mechanisms the SIT group might also be influenced by other molecular mechanisms that GSPE has been shown to modulate, such as, interaction with proteins and enzymes, including the modification of enzymatic activities, binding of receptors and ligands, and DNA transcription factors [38,39]. Moreover proanthocyanidins have also shown to induce the transactivation of some nuclear receptors [40,41].

It has been reported that flavonoids modulate DNA methylation by attenuating the effect of DNMTs, thus inducing a reduction in overall DNA methylation [34,42]. The exact mechanism of *DNMT1* inhibition by flavonoids is still under study but it may take place by, for example, direct enzyme inhibition, indirect enzyme inhibition, reduced *DNMT1* expression and translation, or interaction with methyl-CpG binding domain proteins [42]. Specifically, we checked whether GSPE was affecting the gene expression of *DNMT1*, which is the methyltransferase responsible for maintaining DNA methylation. Our results revealed a tendency for this expression to decrease when GSPE was administered as a pre-treatment, which suggests that this methyltransferase may participate in the regulation of *GLP-1R* methylation in the PRE group. However, the other GSPE treatments, which also showed changes in their methylation pattern, presented no differences in their *DNMT-1* gene expression.

From our study of the putative binding sites in the promoter region of *GLP-1R*, we detected that one putative transcription factor (TF) binding to the promoter could be SP1 in a string of the promoter conserved in the human gene. In the human gene this string has been experimentally proven to be regulated by this TF [43]. SP1 is reported to bind and act through GC boxes to regulate the gene expression of the transcriptional activity of genes involved in most cellular processes [44]. Their down-regulation includes not only interfering directly with the binding of SP1 to their putative DNA binding sites but also promoting the degradation of SP1 protein factors [45]. It has been observed that SP1 facilitates the basal gene expression of *GLP-1R*, which is regulated by negatively acting tissue- and cell-specific cis-regulatory elements. Moreover, Hall et al., who found that the DNA methylation status of *GLP-1R* was modified, suggest that DNA hypermethylation may repress the binding of SP1 to the *GLP-1R* promoter and result in transcriptional silencing [46]. This suggests that, in our case, the hypomethylation of *GLP-1R* in the ileum induced by GSPE may be either favoring SP1 binding to the promoter or inhibiting its regulatory elements, thus promoting the transcription of the gene. However, further functional studies should be conducted to check the specific role of *GLP-1R* DNA methylation in SP1 and gene transcription.

On the other hand, while a GSPE effect on *GLP-1R* mRNA was clearly observed in the ileum, none of the treatments induced any change in the colon with respect to the CAF group. In this case, it appears that the cafeteria diet is responsible for upregulating this gene without being affected by any GSPE treatment. Other studies with GSPE and other tissues found that in rats fed a cafeteria diet and a low dose of GSPE/kg BW for 12 weeks, *GLP-1R* expression in the hypothalamus was unaffected by the cafeteria diet, whereas it was downregulated by the GSPE treatment in comparison with cafeteria-fed rats [47]. Another study with high fat diet (HFD)-induced obese male mice subjected to bariatric surgery analyzed *GLP-1R* to determine whether an altered luminal environment could specifically affect the mucosal expression of this receptor. However, the image analysis revealed no difference in pixel number or expression patterns between the chow diet and HFD groups [48]. These results disagree with ours. Other authors have reported that colonic expression of *GLP-1R* mRNA is significantly upregulated in response to a HFD [49], thus agreeing with our results. Possible reasons for these differences include the use of different species, the composition of the diet, and the surgery to which the animals were subjected. With regard to the effects we observed in the colon, we do not believe that these were caused by changes in the methylation of the *GLP-1R* promoter since there were no significant changes in any of the CpG sites studied, just a tendency to increase methylation in CpG site 2. This pattern has also been found in other studies in which the terminal ileum has presented different methylation patterns in the ascending and sigmoid colon [50,51]. Howell et al. suggested that the different intestinal epithelial cell-specific epigenetic alterations depend at least partly on the stability of such molecular signatures [50]. The molecular mechanisms underlying the regional variations in methylation patterns along the GI tract are not understood. However, the molecules that reach the colon have frequently been metabolized by gut microbiota and are therefore different from those that reach the ileum [52]. Moreover, possible changes in microbiota-derived products may also be responsible. Indeed, analysis of the effect of gut microbiota on *GLP-1* activity revealed that gastrointestinal motility is accelerated while *GLP-1R* expression is suppressed in myenteric neural cells throughout the gastrointestinal tract [27]. Short chain fatty acids are also reported to induce changes in DNA methylation. High exposure to succinate has been related to DNA hypermethylation in vitro [53]. In our case, we found a positive correlation between hypomethylations of *GLP1-R* and succinate in the ileum but a negative correlation between hypomethylation of *GLP1-R* and acetic acid in the colon. The possible effect of acetic acid on DNA methylation was suggested by the administration of acetate and/or other SCFA to prevent body weight gain in male mice with high-fat diet-induced obesity [54]. Moreover, the different profiles of the correlations between the ileum and the colon may be related to the different methylation patterns in these tissues, which may also indicate possible changes in microbiota.

In summary, a 10-day pre-treatment with GSPE (500 mg/Kg bw) induces long-lasting effects on *GLP-1R* gene expression, possibly via a reduction in DNA methylation at the gene promoter in the ileum. Moreover, the DNA hypomethylation pattern changes depending on the moment of GSPE administration in the ileum. These effects were not observed in equivalent treatments administered concomitantly to a cafeteria diet in the colon.

## Figures and Tables

**Table 1:** Primer sets used for pyrosequencing.

Rat	*GLP-1R*	Forward	5′-GTTGAGGGGGAGTTTGGA-3′
		Reverse	5′-ACCCCAAAAATAAAACCTCCAACTCTA-3′
		Sequencing	5′-GGGAGGAGGGTTTTAATG-3′

**Table 2:** Spearman correlation coefficients (rho, ρ) between the methylation different CpG studied in the promoter of *GLP-1* receptor in the ileum and cecal short chain fatty acids considering all the groups together.

Variables	Formic	Acetic	Propionic	Butyric	Valeric	Succinic
Pos. 1	−0.081	0.033	0.192	0.162	0.030	0.284
Pos. 2	−0.064	0.023	0.024	0.229	−0.035	0.381 *
Pos. 3	−0.013	0.098	−0.221	0.346 ^#^	0.096	0.197
Pos. 4	0.113	0.115	−0.093	0.451 *	0.386 *	0.598 *
Pos. 5	−0.028	0.054	−0.222	0.418 *	0.261	0.557 *
Average of Positions	−0.063	0.014	−0.079	0.331 ^#^	0.142	0.487 *

^#^ indicates that the correlation is significant at the 0.1 level (bilateral) and * indicates that the correlation is significant at the 0.05 level (bilateral).

**Table 3:** Spearman correlation coefficients (rho, ρ) between the methylation different CpG studied in the promoter of *GLP-1* receptor in the colon and cecal short chain fatty acids considering all the groups together.

Variables	Formic	Acetic	Propionic	Butyric	Valeric	Succinic
Pos. 1	−0.410 ^#^	−0.448 ^#^	−0.464 ^#^	−0.163	−0.370	−0.379
Pos. 2	−0.170	−0.327	−0.392	0.026	−0.244	−0.525 *
Pos. 3	−0.300	−0.405 ^#^	−0.224	−0.238	−0.370	−0.517 *
Pos. 4	−0.505 *	−0.617 *	−0.380	−0.349	−0.449 ^#^	−0.369
Pos. 5	−0.450 ^#^	−0.550 *	−0.318	−0.416 ^#^	−0.465 *	−0.474 *
Average of Positions	−0.352	−0.486 *	−0.346	−0.216	−0.399 ^#^	−0.424 ^#^

^#^ indicates that the correlation is significant at the 0.1 level (bilateral) and * indicates that the correlation is significant at the 0.05 level (bilateral).

## Supplementary Materials

The following are available online at https://www.mdpi.com/2218-273X/9/12/865/s1, Figure S1: Schematic diagram of the experimental design, Table S1: Cecal short chain fatty acids.
