# Supplementary material for: Long-Lasting Effects of GSPE on Ileal GLP-1R Gene Expression Are Associated with a Hypomethylation of the GLP-1R Promoter in Female Wistar Rats
Source: Biomolecules. 2019 Dec 12;9(12):865. doi: 10.3390/biom9120865 (PMC6995503; doi:10.3390/biom9120865)
Supplement: Supplementary file 1 [file biomolecules-09-00865-s001.pdf]

Supplementary materials

Materials and Methods

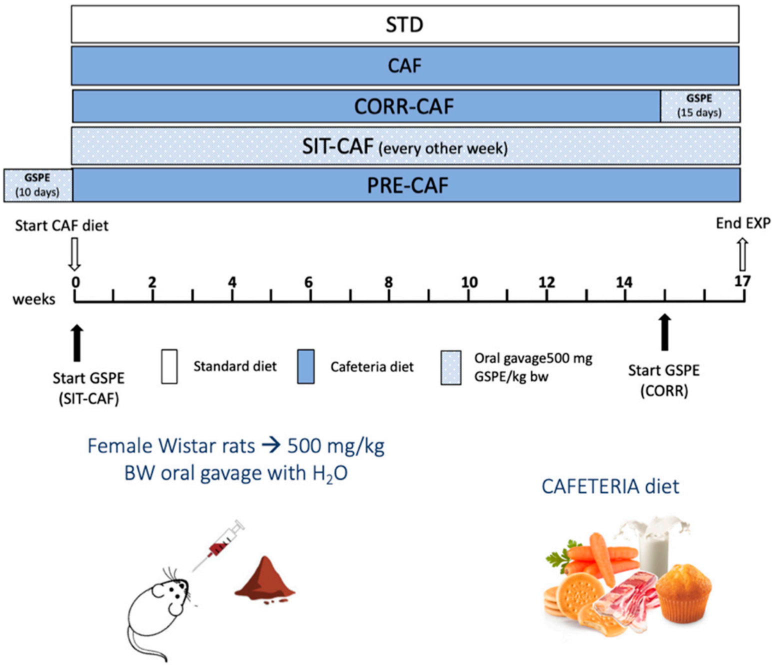

Figure 1. Schematic diagram of the experimental design. STD: standard chow diet; CAF: cafeteria diet; PRE-CAF: rats receiving GSPE (Grape-seed proanthocyanidins) preventive treatment 10 days before the cafeteria intervention started; SIT-CAF: rats receiving GSPE treatment simultaneously and intermittently with the cafeteria diet every other week; CORR-CAF: rats receiving GSPE corrective treatment during the last 15 days of the cafeteria intervention. GSPE: grape seed proanthocyanidin extract.

SCFA quantification

The concentration of SCFA (formic, acetic, propionic, butyric, isovaleric, valeric, lactic an,d succinic) were assayed in cecal content thawed at 4 °C. Briefly, _1 g of sample was added to a screw cap glass tube containing 1 mL ultrapure deionized water (1:1, w/w), and was vortexed vigorously. One hundred microliters of the internal standard 4-methyl-valeric acid, 2 mL of ethylic ether, and 0.5 mL of HCl 37% were added to these samples, and in parallel to 1 mL, a reference solution containing different concentrations of the standard SCFA was added. The tube was vortex mixed for 1 min and centrifuged for 15 min at 3500 Å~ g. Sixty-five microliters of supernatant was used for the derivatization step, performed with 10 µL of MTBSTFA ((N-methyl-N (tert-butyldimethylsilyl)-trifluoroacetamide), Aldrich 375934) at 80 °C for 30 min. Derivatized SCFAs were analyzed using gas chromatography (Agilent 6890-NT, Santa Clara, USA,) coupled with a 30 m Å~ 0.25 mm id, column, with a film thickness of 0.25 micras (Agilent DB-23, Barcelona, Spain) and a flame ionization detector (FID) to determine SCFA concentrations [1]. The carrier gas used was helium. A constant flow mode was used (split 25:1; 30 mL min^{-1} split flow). The column temperature was programmed to gradually increase from 60 to 220 °C during the analysis. In addition, injector port and FID temperatures were fixed at 250 °C. Injection volume was set to 1 µL, and analyses were performed in duplicate [2].

Table S1. Cecal short chain fatty acids.

µmols/g	Acetic	Butiric	Formic	Isobutiric	Propionic	Succinic	Valeric

STD	117.69 ± 8.2	70.61 ± 5.5 *	19.06 ± 1.4	0 ± 0 #	17.07 ± 2.7 #	7.01 ± 0.9	2.48 ± 0.2
CAF	93.72 ± 17.6	19.07 ± 3.6	16.29 ± 1.4	1.58 ± 1.0	37.57 ± 9.8	6.09 ± 1.3	2.02 ± 0.5
SIT	83.73 ± 8.9	12.21 ± 3.0	16.43 ± 1.2	0.20 ± 0.1	31.65 ± 5.2	5.27 ± 1.8	1.04 ± 0.5
PRE	85.75 ± 13.06	21.00 ± 6.01	15.75 ± 1.81	0.24 ± 0.24	30.20 ± 7.95	6.34 ± 1.10	314 ± 0.51
CORR	82.82 ± 21.1	10.50 ± 1.4 *	14.71 ± 2.7	12.15 ± 8.4	64.17 ± 18.8	3.21 ± 0.12	0.74 ± 0.5 #

Values represent mean ± SEM of 6–10 animals per group. *$p \leq 0.05$ versus CAF; # $p \leq 0.1$ versus CAF. Lactic acid was also measured but not found.

1. Rebolé, A.; Ortiz, L.T.; Rodríguez, M.L.; Alzueta, C.; Treviño, J.; Velasco, S. Effects of inulin and enzyme complex, individually or in combination, on growth performance, intestinal microflora, cecal fermentation characteristics, and jejunal histomorphology in broiler chickens fed a wheat- and barley-based diet. *Poult. Sci.* **2010**, *89*, 276–286.
2. Calik, A.; Ergün, A. Effect of lactulose supplementation on growth performance, intestinal histomorphology, cecal microbial population, and short-chain fatty acid composition of broiler chickens. *Poult. Sci.* **2015**, *94*, 2173–82.
